# Acute viral infection results in a PD-1-dependent loss of anti-tumor CD8+ T cell responses: implications for tumor immunotherapy

**DOI:** 10.1186/2051-1426-3-S2-P281

**Published:** 2015-11-04

**Authors:** Frederick Kohlhapp, Erica Huelsmann, Joseph Broucek, Jason Schenkel, Howard Kaufman, Andrew Zloza

**Affiliations:** 1 Rutgers CINJ, New Brunswick, NJ, USA; 2 Rush University, Chicago, IL, USA; 3 University of Minnesota, Minneapolis, MN, USA

## Introduction

Oncolytic viruses are gaining acceptance as a method of tumor immunotherapy, and some studies have reported improved anti-tumor T cell responses after acute pyretic non-oncolytic infections. Therefore, we hypothesized that viral infections may aid in the immunotherapy of cancer.

## Methods

To test this hypothesis, we used the A/H1N1/PR8 mouse model of influenza infection (10,000 pfu, via intranasal administration) prior to or after challenge with B16 melanoma (12,000 - 120,000 cells via intradermal injection in the right flank). Kaplan-Meier curves were used to present animal survival and tumor incidence data and log rank test was used to compare such curves. All statistical analyses were performed using Prism software (v4.0, GraphPad software), and a p value of less than 0.05 was considered to denote statistically significant differences among compared variables.

## Results

Contrary to our hypothesis, we observed that acute influenza infection of the lung promotes distant melanoma growth in the dermis of the flank and results in up to 50% decrease in host survival (P < 0.001). We observed similar findings across a series of different infection and tumor models. In experiments designed to determine whether infection could lead to the emergence of cancer that is otherwise controlled by the immune system, we challenged mice with serial cell numbers of B16 (12,000 and 1,200) at which tumor incidence is reduced (to 60% and 0%, respectively). Here, influenza infection significantly increased tumor incidence for both 12,000 and 1,200 B16 cell challenges to 100% (P < 0.01 and P < 0.001, respectively). Further, using a tumor transplant model we demonstrated that anti-melanoma CD8+ T cells (defined by a congenic marker and/or tetramer) in the context of influenza infection are shunted to the lungs (P < 0.01 vs non-infected mice), where they express high levels of the activation and exhaustion receptor, PD-1 (P < 0.01). Immunotherapy with PD-1 blockade reverses this loss of anti-tumor CD8+ T cells from the tumor and decreases viral infection-induced tumor growth.

## Conclusions

Our findings define a previously unrecognized mechanism of immune suppression in cancer-bearing hosts, namely acute non-oncogenic viral infection. This finding may be important because it raises concerns over patient exposure to acute viral illness, vaccination, and treatment with viral vectors in tumor immunotherapy; and it highlights an unexpected mechanism of PD-1 blockade in the treatment of cancer.

